# The Actions and Feelings Questionnaire in Autism and Typically Developed Adults

**DOI:** 10.1007/s10803-017-3244-8

**Published:** 2017-07-28

**Authors:** Justin H. G. Williams, Isobel M. Cameron

**Affiliations:** Clinical Research Centre, Division of Applied Health Sciences, University of Aberdeen, Royal Cornhill Hospital, Aberdeen, Scotland AB25 2ZH UK

**Keywords:** Autism, Empathy, Motor cognition, Sensorimotor, Questionnaire, Confirmatory factor analysis

## Abstract

Impaired motor cognition may underpin empathy problems in autism. The actions and feelings questionnaire (AFQ), designed to examine individual differences in motor cognition, was completed fully by 1391 adults, of whom 326 reported a diagnosis of an autism spectrum condition (ASC). A confirmatory factor analysis supported a 3 factor model. The AFQ total and ‘feelings’ subscale scores correlated highly with the EQ and ROC curves were similar. Our findings suggest that individual differences in empathic traits are heavily accounted for by variance in mechanisms that serve sensorimotor learning (motor cognition) in relation to emotional states. The AFQ is a self-report tool that provides a valid indicator of autism status in adult populations.

**Electronic supplementary material:**

The online version of this article (doi:10.1007/s10803-017-3244-8) contains supplementary material, which is available to authorized users.

## Introduction

Empathy, is a multi-faceted construct that relies on a variety of processes and is variably defined (Decety 2011). One definition: to understand other people’s emotional states, as well as to experience them vicariously (Baron-Cohen and Wheelwright 2004), covers processes which include emotional contagion, whereby the expression of emotion by one individual is experienced by another (Hatfield et al. 1993), the conscious awareness of this experience (de Vignemont and Singer 2006) and mentalizing; a cognitive mechanism whereby mental states are inferred through the observation of behaviour (Brass et al. 2007).

In recent years, evidence has been accumulating for a “motor cognition” model of empathy (Decety and Meyer 2008; Jackson and Decety 2004); the fundamental unit of this paradigm being action, defined as “movements produced to satisfy an intention towards a specific goal, or in reaction to a meaningful event in the physical and social environments”. Motor cognition includes the processes involved in the perception, recognition and interpretation of action as well as the processes concerned with action preparation and production. Motor cognition theory argues that both emotional contagion and mentalizing rely on the neural mechanisms dedicated to the perception and enactment of actions. This model is particularly suited to understanding the basis for processing the social information that constitutes emotional communication, as well as the first person experience of those emotions. A motor cognitive model of empathy suggests that empathy relies on a “simulation” theory of mind (Gordon 1996), whereby an observer re-creates the mental states generating emotionally communicative actions in someone else, by observing those actions, and enacting them in the imagination. Firstly, perceived actions elicit neural activity in the observer in those brain areas that would serve the first person sensation of the stimuli which lead to those same behaviours. For example, observing pain being inflicted on others elicits activity in somatosensory cortex serving pain perception, and consequently, the vicarious experience of pain (Avenanti et al. 2005; Lamm et al. 2011; Singer et al. 2004). Similarly, observing the expression of disgust by others elicits activity in the insula, an area that serves the first person experience of disgust (Keysers and Gazzola 2007).

Empathic function may be considered to operate in a hierarchical fashion. In Decety and Mayer’s (2008), motor cognition model of empathy, motoric and affective (non-reflexive, automatic and unconscious) resonance mechanisms at the bottom, are subject to top-down control by metacognitive, conscious and intentional control mechanisms at a higher level. Kilner et al. (2007) suggest that goal-directed actions exist within hierarchies (each goal is part of a larger goal) and predictive-coding models for actions also function within hierarchies with lower levels being controlled by higher levels. Therefore, the degree to which another person’s action is perceived empathically (and consciously), will depend firstly on vicarious experience, but then secondly, on modulatory mechanisms that serve to encode goal-directed actions at an abstract level, such as where the goal is inferred from social context. Vicarious responses to others’ displays of emotion may reflect emotional contagion, but not necessarily with awareness, and are likely to be subject to control by sensori-motor feedback and control mechanisms (Hatfield et al. 2014; Lee et al. 2013). Hess and Fischer (2013) argue that social communication develops through the mimicry of contextualised emotion signals (empathy) rather than direct copying, and is therefore in keeping with the Decety and Meyer (2008) model whereby the learning of emotionally communicative signals is developed under top-down control.

Autism is partly defined by impairments in socially communicative and reciprocal social behaviours, and so impaired empathy is a central feature. However, this does not mean that all aspects of empathy are affected. Impairments in the development of hierarchical control of predictive coding mechanisms, may result in impairments of goal-inference. This could result in enhanced emotional contagion at lower levels, but impaired understanding (Gu et al. 2015). Whilst some earlier studies argued for impaired emotional contagion for smiling (McIntosh et al. 2006; Oberman et al. 2009), yawning (Senju et al. 2007) and pain perception (Minio-Paluello et al. 2009) in autism, more recent studies find it to be intact or even enhanced (Fan et al. 2014; Gu et al. 2015; Hadjikhani et al. 2014; Rogers et al. 2007; Senju et al. 2009). Similarly, emotion recognition is only weakly affected in autism (Law Smith et al. 2010), though larger group differences may emerge when facial expression stimuli are dynamic rather than static (Sato et al. 2012; Yoshimura et al. 2015).

One possible way that empathy may be disrupted in autism is through its dependence on motor cognition and arguments have been made that impaired motor cognition underlies ASD (e.g. Rogers and Pennington 1991; Williams et al. 2001; Williams 2008). Impaired motor cognition may also result in other features of autism that rely on hierarchical control of feedback-dependent, sensorimotor learning. These include: sensory symptoms (Pellicano and Burr 2012), poor imitation skills (Edwards 2014), poor learning of gesture and a reduced range of facial expressions (Williams et al. 2001; Williams 2008). Many of the diagnostic features of autism reflect impaired use of action in social communication and reciprocity. Items reflecting gesture and facial expression make up a substantial proportion of the algorithmic items in diagnostic instruments such as the autism diagnostic observation schedule (ADOS; Lord et al. 2000) and autism diagnostic interview-revised (ADI-R; Lord et al. 2000; Rutter et al. 2000). The association between deficits in use of communicative actions and empathic impairments occurring closely together in autism raises the possibility of a causal relationship between the two systems.

Assessment of non-verbal communication skills and motor cognition is therefore an important aspect of autism assessment as well as a variety of other mental health problems. The importance of non-verbal communication has been recognised in its making up of two constructs within the research domain criteria (RDoC) strategy (Morris and Cuthbert 2012); http://www.nimh.nih.gov/research-priorities/rdoc/rdoc-constructs.shtml#production_nonfacial_communication. Nevertheless, paradigms for its research remain at early stages of development, perhaps because of the complex challenges concerned with the measurement of behaviours which have multiple degrees of freedom. As a starting point, Williams et al. (2015) developed a self-report measure of reliance on gesture and action imagery in social communication and daily life (The Actions & Feeling Questionnaire—AFQ), designed to assess individual differences in motor cognition. The questionnaire showed strong internal consistency, good test–retest reliability, a plausible two-factor structure (production and perception) and demonstrated convergent validity with the brief version of the empathic quotient (EQ); a self-report measure of empathic traits (Muncer and Ling 2006). The authors suggested that because the AFQ was self-reported, it reflected a self-awareness of action. This was supported by a finding that AFQ score correlated with activity in somatosensory cortex during imitation (because other evidence suggests that that activity in somatosensory cortex during action-observation occurs when attention is paid towards actions, and levels of action-awareness are increased). They suggest that much of the individual variability in empathic traits is shared by variability in action-awareness. However, it was also the case that a significant proportion of the items refer to experiencing feelings when they are expressed as actions by others, and it is argued that somatosensory cortex is employed when perceiving both one’s own and other’s feelings (Damasio and Carvalho 2013).

This initial study suggested that the AFQ might be a potent discriminator for differentiation between populations with and without an autism spectrum condition (ASC). As such it could be a useful screening tool in adult and adolescent populations. It may also prove informative in dissecting different aspects of empathic impairment, though a complementary question that remains to be answered is whether it taps into a different construct to the EQ or the same one. Our initial study was based on a modest sample (n = 256). The purpose of the current study was to assess the psychometric properties in a new and larger sample that included participants with and without an ASC.

## Methods

### Questionnaires

The AFQ (Williams et al. 2015) consists of 18 items designed to measure motor cognition. Each item requires a level of agreement which ranges between strongly agree, slightly agree, slightly disagree and strongly disagree. Items are scored as 3, 2, 1 and 0 respectively and are summed to give a total score between 54 and 0. Higher scores are purported to be indicative of better motor cognition. Some items require reverse scoring.

The 15 item EQ (Muncer and Ling 2006) is a measure of empathic aptitude. Each item requires a level of agreement which ranges between strongly agree, slightly agree, slightly disagree and strongly disagree. Items are scored as 2, 1, 0 or 0 respectively and are summed to give a total score between 30 and 0. Higher scores are purported to be indicative of better empathic aptitude.

### Procedure

The study was approved by the institutional ethics review board. The EQ and AFQ questionnaires were brought together and administered on-line. It was made optionally anonymous. Participants could provide contact details if they wished to be informed of the findings. The questionnaire was made available on a web-site managed by SurveyMonkey and the link was circulated using social networking and other e-mail lists. This included the database of volunteers registered with the Cambridge Autism Research Centre (ARC) (https://autismresearchcentre.net/) as having been diagnosed with an autism spectrum condition. Therefore membership of the ASC group is made through participants reporting that they have been given a clinical diagnosis of an autism spectrum condition.

### Confirmatory Factor Analysis of the AFQ

We sought to assess the dimensionality of the theoretical construct “motor cognition” in the AFQ. Confirmatory factor analysis (CFA) was performed to allow for the assessment of several plausible models and their relative fit to be considered. This feature of CFA has an advantage over exploratory factor analysis (EFA) as it enables the testing of the relative “goodness of fit” of varyingly constrained models (Byrne 2005). CFA with maximum likelihood (ML) estimation was conducted using IBM SPSS AMOS 23.

Initially it was hypothesised that motor cognition is unifactorial in nature. In considering competing models, it is usual to test the fit of a general model which represents the most parsimonious approach (Crawford and Henry 2004). Model 1a, a single factor model allowing no correlated error, was first assessed. A further single factor model, Model 1b, was assessed whereby measurement error associated between specified variables was permitted to be correlated. In this model, the correlated error was postulated a priori. Jöreskog and Sörbom (1993) make the case for including correlated error where there is a rationale to do so. We hypothesised likely correlated error between three pairs of items:

i. “Q5: In my mind’s eye, I often see myself doing things” with “Q9: I often imagine myself performing common actions”
ii. “Q14: I move my hands a lot when I speak” with “Q15: I get animated when I am enthusiastic in conversation”
iii. “Q11: When I recall what someone said to me, I have to think hard to remember their facial expression at the time” with “Q16: I can easily bring to mind the look on someone’s face when I remember telling them something”.

Secondly, we considered motor cognition as bipartite, consisting of: perception and production factors, as suggested by the preliminary principal components analysis (PCA) previously reported by Williams et al. (2015). As such, Model 2a (a two-factor model allowing no correlated error) and Model 2b (a two-factor model allowing pre-specified correlated error) were assessed. In this latter, and subsequent models with correlated error permitted, correlations were only permitted if the pairing of items fell within the same proposed factor. Thirdly, a model was postulated in which the motor imagery items were separated and constituted a third factor. We called these three factors feelings, imagery and animation. We postulate the plausibility of such a model in terms of its alignment to the theoretical concept of motor cognition (Jackson and Decety 2004; Decety and Meyer 2008) with such a model differentiating between awareness of actions expressing own and others’ emotions (“feelings”), motor imagery (“imagery”) and expressed actions (“animation”). As with the unifactorial and bipartite models, this tripartite model was assessed without correlated error permitted (Model 3a) as well as with the pre-specified correlated error permitted (Model 3b).

Global assessment of fit of the models was determined by assessing the following: the *Χ* ^*2*^ statistic, the comparative fit index (CFI), the standardised root mean square residual (SRMR) and the root mean square error of approximation (RMSEA). An adequately fitting model may be indicated by a CFI ≥ 0.93; SRMR < 0.08 and RMSEA < 0.06 (Hu and Bentler 1999).

Further psychometric properties were then assessed on the adopted (best fitting) model.

### Internal Consistency

The internal consistency of the AFQ was examined to gauge the extent to which items in the total scale and any adopted subscales gave consistent responses. Cronbach alphas between 0.7 and 0.9 were considered acceptable. Additionally, item-total correlations were computed and considered to be acceptable where values were >0.3 (Everitt 2002).

### Convergent Validity

It was hypothesised that motor cognition is strongly associated with empathy aptitude therefore convergent validity was examined by computing Pearson correlation coefficients of the AFQ with the EQ. Subscales derived from the best fitting CFA model were also correlated with the EQ.

### Predictive Validity of the AFQ

To assess whether the AFQ score (and emergent subscales) could predict group membership, it was hypothesised that participants who indicated having an ASC would have lower scores than participants who indicated they had no ASC (but did have a first degree relative with an ASC) who in turn would have lower scores than participants who had neither an ASC nor a first degree relative with an ASC. However, mean scores showed no significant differences between those with no diagnosis of ASC, according to whether or not they had a first degree relative with ASC [with 1st degree relative with ASC: n = 37; mean (SD): total AFQ = 33.8 (7.39), feelings = 15.86 (4.38), imagery = 5.16 (2.63), animation 9.57 (2.87); without ASD or 1st degree relative with ASC: total = 32.14 (7.46); feelings = 16.49 (3.53), imagery = 5.54 (2.63), animation = 9.57 (3.15)]. Furthermore, the numbers of responses from affected 1st degree relatives was low (n = 37). Therefore for subsequent analysis, we collapsed groups and confined ourselves to examining group status as, ‘yes’, ‘no’ or ‘not sure’. Therefore, group effects for individual items were assessed by conducting a Χ^2^ test on each individual item, testing the null hypothesis that numbers of responses for each score (0–3) would be the same across scores for all groups’ statuses (yes, no and ‘not sure’ for ASC status). Table 7 shows that all items demonstrated a significant effect of group but that this was greatest for the feelings items and lower for the imagery items. Given the differential composition of the ASC and non-ASC groups according to sex, it was possible that sex differences confounded effects of ASC status. To investigate this, data were assessed for normality by appraisal of histogram following which we conducted a multivariate analysis with between-subject factors of sex (male vs. female) and ASC status [ASC (n = 324) vs. No ASC or 1st degree relative with ASC (n = 599)]. It was also hypothesised, given results from Williams et al. (2015), that among participants with no ASC, women would have higher scores than men. Independent T-test and effect sizes were calculated (Table 7).

### Sensitivity and Specificity of the AFQ

Receiver operating characteristics (ROC) curves were used to determine the optimal cut off values of the AFQ, AFQ subscales and EQ for detecting participants with a diagnosis of an ASC. The sensitivity, specificity, positive predictive value (PPV), negative predictive value (NPV), likelihood ratio for a positive test result (LR+) and likelihood ratio for a negative results (LR−) of the scales were then calculated. In choosing an optimal cut off value, there is always a trade-off between sensitivity and specificity. A cut off value with sensitivity and specificity ≥80% was considered acceptable. For these analyses, the sub-group of participants with diagnosis “unknown” were excluded.

## Results

### Participant Characteristics

Of 1707 questionnaires that were initiated, we retained those questionnaires where there was a complete set of responses to the AFQ and participants met our inclusion criteria of being aged 16 years or over. 1391 (81.5%) responses were retained for further analysis. Of those individuals reporting a diagnosis of ASC, 13.2% returned incomplete AFQ’s vs. 15.3% of those reporting no diagnosis. Males constituted 25.7% of those questionnaires deemed incomplete vs. 30.0% of those retained. Three hundred- and twenty-six (23.4%) participants indicated that they had an ASC. Of those, 303 (92.9%) indicated that they had participated in the research via the Cambridge Autism Research Centre. Table 1 documents participant characteristics within ASC status. It can be seen that the groups differed significantly in terms of all characteristics, including sex. Age was also found to correlate weakly (though with a high level of statistical significance) with AFQ in controls but not in those with ASC (see Table 2). Interestingly, this was most evident in the Imagery sub-scale.

**Table 1:** Participant characteristics

Characteristic	Autism spectrum condition (ASC) status				
	ASC N = 326	AFQ total mean (SD)	No. ASC N = 792	AFQ total mean (SD)	Unknown N = 273
Median age (interquartile range**)**	47 (33, 56)		43 (29, 54)		47 (37, 55)
Male sex: n (%)	146 (45.3)	Male: 20.1 (6.71)	212 (26.9)	Male: 29.5 (8.03)	54 (19.9)
		Female: 23.0 (7.9)		Female: 34.6 (6.67)	
English first language: n (%)	260 (80.5)	Yes: 21.1 (7.69)	693 (86.6)	Yes: 33.2 (7.45)	246 (91.4)
		No: 23.5 (6.56)		No: 33.6 (7.25)	
Main activity n (%)					
Student	40 (12.3)	20.35 (6.34)	165 (20.9)	35.56 (6.55)	35 (12.9)
Employed	147 (45.4)	22.58 (7.00)	491 (62.2)	33.12 (7.25)	179 (65.8)
Seeking work	21 (6.5)	23.45 (8.04)	5 (0.6)	30.4 (9.91)	8 (2.9)
Housework	7 (2.2)	23.33 (6.28)	34 (4.3)	33.13 (5.48)	20 (7.4)
Retired	39 (12)	21.75 (6.90)	76 (9.6)	30.02 (8.93)	17 (6.3)
Other	70 (21.6)	19.56 (9.06)	18 (2.3)	30.83 (7.70)	13 (4.8)
Education level n (%)^3^					
Still at school	7 (2.2)	20.5 (4.24)	28 (3.5)	36.4 (5.63)	2 (0.7)
Student (college)	12 (3.7)	24.7 (8.29)	6 (0.8)	32.8 (6.62)	0
Undergraduate student (university)	20 (6.2)	19.5 (7.13)	101 (12.8)	35.4 (6.18)	23 (8.5)
Postgraduate student (university)	9 (2.8)	18.8 (8.26)	35 (4.4)	36.4 (7.96)	13 (4.8)
Minimum age school leaver	42 (13.0)	21.4 (5.59)	55 (7.0)	29.2 (7.64)	20 (7.4)
Completed college	63 (19.4)	21.9 (9.23)	105 (13.3)	32.8 (6.82)	55 (20.3)
Completed university (graduate)	67 (20.7)	21.7 (7.57)	201 (25.5)	33.1 (7.52)	68 (25.1)
Completed university (postgraduate)	104 (32.1)	21.9 (7.17)	258 (32.7)	33.0 (7.50)	90 (33.2)

Age was positively skewed, as such medians and IQR are presented. Kruskal–Wallis test (KWT) for effect of group status on age: p < 0.01. Chi-square tests for effect of group on first language, main activity, and education level: p < 0.001. KWT for effects of main activity on AFQ: p = 0.391 for ASC; p < 0.001 for No ASC. KWT for effects of Education level on AFQ: p = 0.461 for ASC; p < 0.001 for no ASC

**Table 2:** Non-parametric correlations between age and AFQ scores

ASC status	Total	Feelings	Imagery	Animation
No				
Spearman’s R	−0.172	−0.011	−0.250	−0.141
p	<0.001	0.75	<0.001	<0.001
n	793	819	826	825
Yes				
Spearman’s R	0.03	0.074	−0.103	0.029
p	0.592	0.17	0.055	0.595
n	328	342	346	345

### Confirmatory Factor Analysis

**Fig. 1:**
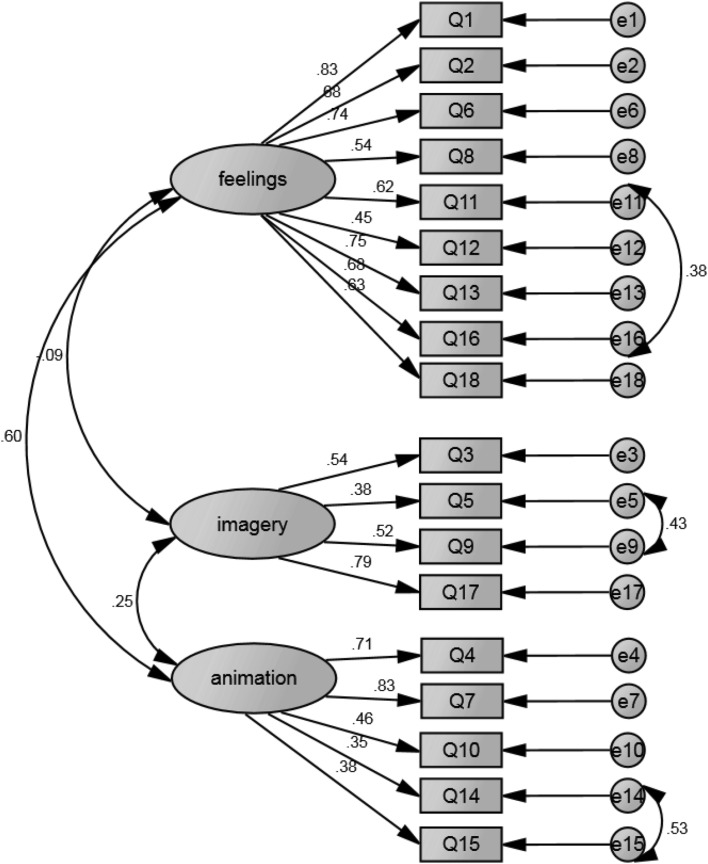
Graphical representation of actions and feelings questionnaire (AFQ) 3 factor model with correlated error

Table 3 presents the fit statistics for the CFA models. The most parsimonious model (Model 1a) can be seen to be poorly fitting. The CFI is lower than acceptable and the SRMR and RMSEA have values that are too high. Permitting the pre-specified correlated error (Model 1b) improves the model slightly but not to an acceptable level. The empirically derived two factor model also shows a poor fit both without (Model 2a) and with (Model 2b) pre-specified correlated error permitted. The three factor model (Model 3a) exhibits improved fit statistics however it is still poorly fitting. When the pre-specified correlated error is permitted (Model 3b) the fit statistics approach that of a well-fitting model. Whilst the CFI does not exceed the 0.93 cut-off, it approximates this value. The value of SRMR is acceptable and the RMSEA is close to the accepted cut-off. As such, the three-factor model with correlated error permitted may be considered as exhibiting a reasonable fit (Fig. 1).

**Table 3:** Fit indices for CFA models of the AFQ (best fitting model in bold)

Model	*Χ* ^b^	*df*	CFI^a^	SRMR^b^	RMSEA^c^	90% CI^d^
1a Single factor	3194.4	135	0.653	0.1145	0.128	0.124–0.132
1b Single factor, correlated error (CE) permitted	1983.5	131	0.790	0.0986	0.101	0.097–0.101
2a Two factor model	2548.5	134	0.726	0.1130	0.114	0.110–0.118
2b Two factor model, correlated error (CE) permitted	1548.0	131	0.839	0.0965	0.088	0.084–0.092
3a Three factor model	1636.8	132	0.829	0.0790	0.091	0.087–0.095
3b Three factor model, correlated error (CE) permitted	**865.7**	**129**	**0.916**	**0.0721**	**0.064**	**0.060–0.068**

^a^Comparative fit index, values ≥ 0.93 indicates a well-fitting model;

^b^Standard root mean squared residual, values < 0.08 indicate a well-fitting model; and

^c^Root mean Square Error of Approximation, values < 0.06 indicate a well-fitting model; ^d^Confidence intervals

### Internal Consistency

Cronbach’s alpha for the total scale was 0.84, for feelings 0.87, for imagery 0.69 and for animation was 0.71. By comparison, the Cronbach alpha for the EQ was 0.90. Three items had item-total correlations <0.3 to the total AFQ (Q3, Q9 and Q17) and all measured “imagery”.

### Convergent validity

**Fig. 2:**
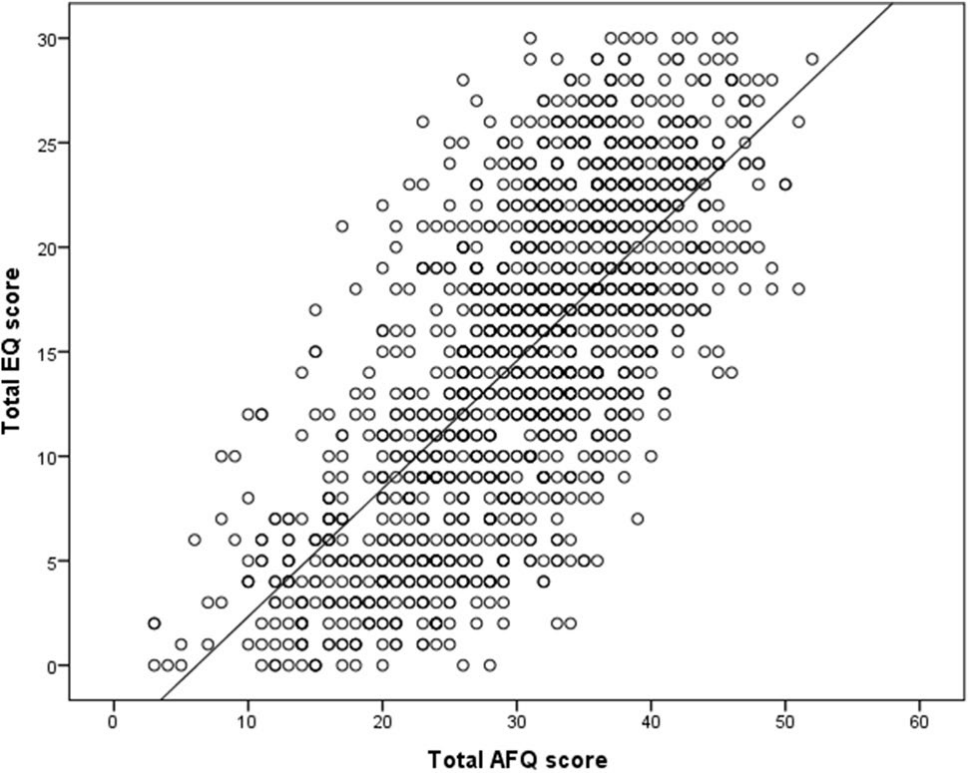
Scatterplot of AFQ with EQ

Table 4 presents the Pearson correlations of the total AFQ and the AFQ subscales with the EQ. It can be seen that the total AFQ had a moderate to large correlation with the EQ which is mainly explained by the Feelings subscale. The Animation subscale has a small to moderate correlation with the EQ and the Imagery subscale would appear not to correlate at all with the EQ. The linear relationship between the AFQ and the EQ is demonstrated in Fig. 2.

**Table 4:** Pearson correlation coefficients of AFQ with EQ

Correlated variables	Autism spectrum condition (ASC) status			
	ASC N = 303	No ASC N = 758	Unknown N = 256	All N = 1317
*r* ^a^ of Total AFQ with EQ	**0.445**	**0.602**	**0.539**	**0.717**
*r* of AFQ Feelings with EQ	**0.567**	**0.724**	**0.642**	**0.810**
*r* of AFQ Imagery with EQ	−0.109	0.004	−0.080	−0.078
*r* of AFQ Animation with EQ	**0.259**	**0.460**	**0.427**	**0.522**

^a^Pearson correlation coefficient; significant at the 0.01 level (two-tailed) are shown in bold

### Predictive Validity

**Fig. 3:**
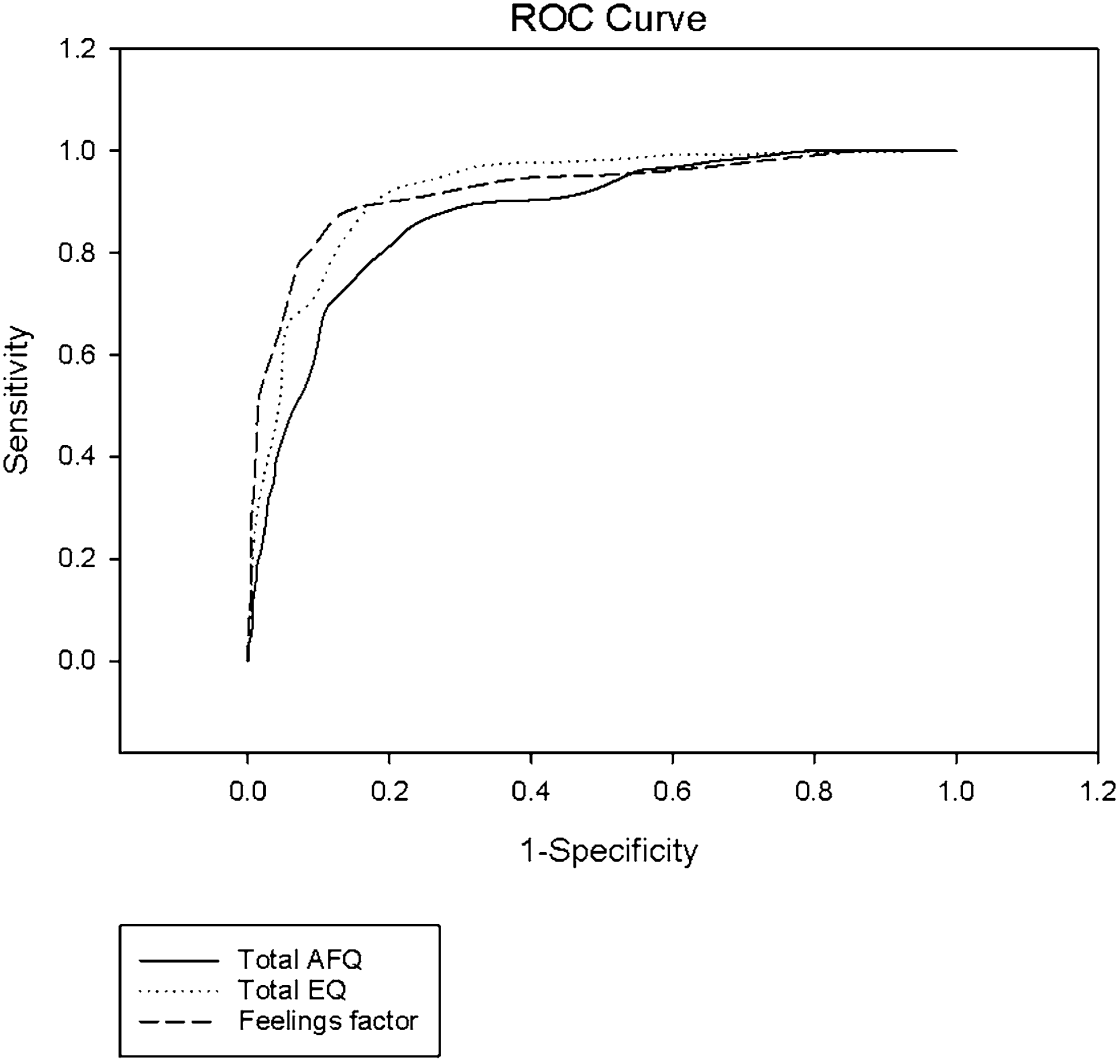
Receiver operating characteristics (ROC) curve of AFQ total, AFQ feelings subscale and EQ

It can be seen from Tables 5 and 6 and Fig. 3 that participants without ASC score much higher on the total AFQ and AFQ Feelings than people with an ASC. A smaller effect of sex is observed, and a statistically significant interaction of very small effect. Less magnitude is observed for the Imagery and Animation subscales but ASC group differences are still significant.

**Table 5:** Mean scores by sex and ASC status

ASD	Sex	Mean	SD	N
Total AFQ				
Yes	Male	20.04	6.83	137
	Female	22.56	7.51	164
No	Male	29.61	7.98	207
	Female	34.64	6.57	547
Total	Male	25.80	8.88	344
	Female	31.85	8.49	711
Total EQ				
Yes	Male	5.43	4.02	137
	Female	6.84	4.46	164
No	Male	14.58	6.06	207
	Female	18.70	5.95	547
Total	Male	10.94	6.97	344
	Female	15.96	7.53	711
Feelings				
Yes	Male	7.45	4.36	137
	Female	7.82	4.61	164
No	Male	15.08	3.98	207
	Female	16.88	3.40	547
Imagery				
Yes	Male	5.99	2.83	137
	Female	6.32	2.90	164
No	Male	5.25	2.64	207
	Female	5.70	2.56	547
Animation				
Yes	Male	5.88	2.81	137
	Female	7.73	3.23	164
No	Male	7.82	3.09	207
	Female	10.18	2.78	547

In participants without ASC, female scores are higher than male scores with moderate effect sizes for all but the imagery subscale (Table 6). Group effects for individual items were assessed by conducting a *X* ^*2*^ test on each individual item, testing the null hypothesis that numbers of responses for each score (0–3) would be the same across scores for all groups’ statuses (yes, no and ‘not sure’ for ASC status). Table 7 shows that all items demonstrated a significant effect of group but that this was greatest for the feelings items and lower for the imagery items.

**Table 6:** Between-subject factors of sex [male (n = 344) vs. female (711)] and ASC status [ASC (n = 301) vs. No ASC n = 754)] those who responded ‘unsure’ for ASC status have been excluded

	df	F	p	Partial η^2^
ASC status				
Total AFQ	1	470.50	<0.001	0.309
Total EQ	1	716.28	<0.001	0.405
Feelings	1	934.74	<0.001	0.471
Imagery	1	12.85	<0.001	0.012
Animation	1	111.84	<0.001	0.096
Sex				
Total AFQ	1	57.20	<0.001	0.052
Total EQ		49.55	<0.001	0.045
Feelings	1	15.80	<0.001	0.015
Imagery	1	4.35	0.037	0.004
Animation	1	103.35	0.000	0.090
ASC × sex interaction				
Total AFQ	1	6.28	0.012	0.006
Total EQ	1	11.87	0.001	0.011
Feelings	1	6.97	0.008	0.007
Imagery	1	0.09	0.765	–
Animation	1	1.52	0.218	–

a. Total AFQ: adjusted R Squared = 0.396. b. Total EQ: adjusted R Squared = 0.484. c. Feelings: adjusted R Squared = 0.519. d. Imagery: adjusted R Squared = 0.012. e. Animation: adjusted R Squared = 0.222

### Sensitivity and Specificity of the AFQ

Areas under the ROC curves, with related confidence intervals, are shown in Table 8. Largest areas under curves were evident for the AFQ feelings subscale and the EQ, followed by the AFQ total scale (Fig. 3). The AFQ imagery and animation subscales show lower areas under the curve. As such, the AFQ total, EQ total and AFQ feelings subscale were the focus for further analysis of diagnostic test accuracy. Tables 9 and 9 shows where the best trade-off between sensitivity and specificity was reached for each scale (as indicated in bold).

**Table 7:** The 18 item AFQ. chi-squared and p-values values illustrate magnitude of group effect on ratings for individual items

Item		Factor	Chi-squared	p
1.	I tend to pick up on people’s body language	Feelings	588	<0.0001
2.	To understand someone I rely on his or her words rather than their expression or gesture^a^	Feelings	440	<0.0001
3.	To make sense of what someone else is doing, I might copy his or her actions	Imagery	76.7	<0.0001
4.	Music that I like makes me want to dance	Animation	116	<0.0001
5.	In my mind’s eye, I often see myself doing things	Imagery	21.7	0.001
6.	If talking on the phone, I am sensitive to someone’s feelings by the tone of their voice	Feelings	514	<0.0001
7.	If others are dancing I want to join in	Animation	265	<0.0001
8.	My body movements do not tend to reflect the way I feel^a^	Feelings	322	<0.0001
9.	I often imagine myself performing common actions	Imagery	17.1	0.01
10.	I would consider myself to be a “touchy-feely” person	Animation	171	<0.0001
11.	When I recall what someone said to me, I have to think hard to remember their facial expression at the time^a^	Feelings	425	<0.0001
12.	I rely on seeing how a person looks me in the eye to gauge what they really feel	Feelings	274	<0.0001
13.	I wouldn’t tend to know what someone was feeling like if they did not say^a^	Feelings	469	<0.0001
14.	I move my hands a lot when I speak	Animation	44.6	<0.0001
15.	I get animated when I am enthusiastic in conversation	Animation	28.8	<0.0001
16.	I can easily bring to mind the look on someone’s face when I remember telling them something	Feelings	384	<0.0001
17.	Acting things out helps me to understand them	Imagery	27.4	<0.0001
18.	Watching someone’s body language is not a good way to judge their feelings^a^	Feelings	299	<0.0001

^a^Negatively scored item

**Table 8:** Area under the ROC curve of AFQ total, AFQ feelings, AFQ imagery, AFQ animation and EQ

Scale	Area under ROC curve	95% CI^a^
AFQ	0.873	0.850–0.896
AFQ feelings	0.923	0.905–0.942
AFQ imagery	0.441	0.402–0.481
AFQ animation	0.723	0.690–0.756
EQ	0.923	0.906–0.940

^a^Confidence intervals

**Table 9:** AFQ, AFQ Feelings Subscale and EQ: discriminatory performance of detecting an autism spectrum condition (ASC)

Scale	% Sensitivity	% Specificity	% PPV^a^	% NPV^b^	LR+^c^	LR−^d^
AFQ total						
<26	71	86	68	88	5.24	0.33
<27	76	83	65	90	4.51	0.28
**<28**	80	80	62	91	3.95	0.25
<29	84	77	60	92	3.64	0.21
AFQ Feelings						
<11	78	92	80	91	9.53	0.24
<12	82	90	77	92	8.04	0.20
**<13**	86	87	73	94	6.42	0.16
<14	90	81	66	95	4.67	0.13
EQ total						
<10	79	88	73	91	6.74	0.25
<11	83	86	70	93	5.97	0.19
**<12**	89	83	67	95	5.18	0.14
<13	93	79	63	96	4.34	0.09

Scores in bold indicate optimum cut-off points

^a^Positive predictive value

^b^Negative predictive value

^c^Likelihood ratio for a positive result (the likelihood of having a disease, as opposed to not having the disease, having tested positive for it)

^d^Likelihood ratio for a negative result (the likelihood of having a disease as opposed to not having that disease having tested negative for it)

## Discussion

We found the actions and feelings questionnaire to correlate highly with the empathy quotient and to perform similarly to it in differentiating between sexes. In differentiating between populations of adults with and without autism, the total AFQ score also showed a similar effect size of ASC status to EQ (Cohen’s d: total AFQ = 0.65; EQ = 0.68).

The relationship can be better understood through examination of the factor analysis. We found that a three factor model provided the best fit for our data. The first factor contained those items which asked about the sensitivity to other people’s emotions through the observation of their non-verbal behaviour, as well as links between one’s own feelings from own actions. This feelings subscale provided a strong indicator of autism status (effect sizes [η2]: Feelings subscale = 0.527; EQ = 0.477). With respect to classification accuracy, the feelings subscale score showed properties similar to the EQ with sensitivity of 86% at specificity of 87%. A possibility that may limit the value of the measure as an indicator of diagnostic status, is that scores are affected by level of education. Table 1 shows a breakdown of AFQ total scores by ASD status. This shows small but significant effects educational level, in those without ASC. However, AFQ total scores were similar for those for whom English is their first language or not, and also for college and university students and graduates. Therefore, the limited demographic data does not suggest that English language ability or educational level influences scores. Table 1 also shows associations between AFQ scores and ASC status, with main activity. Participants with an ASC reported lower levels of employment (62.2 vs. 45.4%), corresponding to reports showing reduced employment among those with autism (e.g. Hedley et al. 2016). Of those without an ASC, those reporting themselves to be employed, students or occupied by housework had slightly higher AFQ scores. Whilst this could be due to effects of motor empathy on occupational functioning, it may also be due to differential sampling by age and sex.

The second factor, which we termed “imagery” contained items referring to the imagination of actions (motor imagery), whilst the third factor asked people how animated they tend to be in expressing themselves, whether through gesture in communication or dancing to music. Animation showed moderate associations with EQ and sex and highly significant group effects. Imagery showed a small effect of sex but curiously an association with autism, in the opposite direction expected. Although this was only a small effect, it was still highly significant in view of the large sample size. This may be due to enhanced visuospatial abilities which are well recognized in autism (Shah and Frith 1993), that may be used to compensate for other impairments, as has been suggested with language (Kana et al. 2006). Interestingly, imagery was the subscale that most correlated with age, albeit inversely and weakly. Given that this study was carried out in adults, this finding is hard to interpret, but a hypothesis for future study is that typical motor cognitive development is characterized by a decreasing reliance on lower-level sensorimotor representation, including motor imagery and animation, but that this is delayed in autism.

Our study therefore supports a hypothesis of strong overlap between the empathy construct as measured by the EQ, and motor cognition, but also some clear dissociation. The internal consistency of the AFQ was good (Cronbach’s alpha = 0.84) but the imagery factor correlated poorly with the feelings factor and EQ, and was poor at discriminating between ASC and typical populations. This suggests that motor cognition is not uniformly impaired in autism but rather, particularly when it is appropriated for reading emotion. Notably the item referring to perception of own feelings was also closely associated with the ‘feelings’ factor indicating that it is not just about reading the feelings of others.

Williams et al. (2015) suggested that AFQ was a measure of action-awareness and emotion-awareness, given the argument that emotions are ‘embodied’ by actions (Niedenthal 2007) and their finding of an association between AFQ score and activity in somatosensory cortex during imitation of emotional expressions. Our findings are therefore consistent with diminished emotion awareness in ASD. Diminished levels of emotional awareness are well recognised in ASD, and it has been associated with high levels of alexithymia, which is a disorder characterised by an impaired ability to recognise and identify emotions in one’s self (Brewer et al. 2015; Cook et al. 2013). Alternatively, the correlation between AFQ score and emotion-awareness could occur through common dependence on an interoceptive function. It is debated whether individual differences in emotion-awareness in autism relate to differences in interoceptive ability (Brewer et al. 2015; Garfinkel et al. 2016; Quattrocki and Friston 2014; Shah et al. 2016), which is hypothesised on the basis of the long-standing premise that emotional states have their origins in bodily states, and reflect cognitive evaluation of physiological changes (Lange and James 1922; Seth 2013). However, the empirical evidence from studies of interoception in autism is inconsistent (DuBois et al. 2016), and it is suggested that separable aspects of interoceptive ability such as subjective awareness and accuracy are differentially affected (DuBois et al. 2016; Garfinkel et al. 2016).

A popular distinction often made in the literature is between “cognitive” or “emotional” aspects of empathy, which raises the question as to whether the construct of “motor empathy” which the AFQ aims to measure, fits with respect to this division. Cognitive empathy is generally defined as an ability to “understand” others’ behaviour (e.g. Baron-Cohen and Wheelwright 2004), and is used to refer to seemingly non-emotional, cognitive functions such as perspective taking, metarepresentation, inferential learning and understanding of false belief. It is thought to be served by temporoparietal junction and medial prefrontal cortex (Saxe 2006). In contrast, emotional empathy is concerned with the vicarious experience of emotion and pain, and is thought to be served mainly by somatosensory cortex, anterior cingulate and insula (de Vignemont and Singer 2006). A number of approaches have sought to attribute individual differences in empathic traits to variation in either cognitive or emotional components of empathy. However, questionnaire studies with the EQ have indicated a single factor model best explains its patterns of variance (Allison et al. 2011) and the EQ correlates highly with the Toronto empathy questionnaire (TEQ) which purports to measure “Emotional Empathy” (Spreng et al. 2009). Possibly, sex differences might occur for cognitive but not emotional empathy, as studies of facial mimicry and emotional contagion have found none of the sex differences that are evident when measuring more cognitive empathic traits (Hatfield et al. 2014), which though mixed, suggests a female advantage appears to emerge with age, possibly dependent upon culture and learning (Devine and Hughes 2016).

A motor cognition framework offers a slightly different perspective. The cognitive/emotional empathy divide appears to correspond to Decety and Meyer’s (2008) distinction between “top-down” and “bottom-up” elements in their motor cognitive model of empathy. Emotional empathy is relatively automatic and reactive, whilst cognitive empathy is under more intentional control.

Sensorimotor learning tasks range from being almost purely an exercise in visuospatial calculation with a minimal emotional component (e.g. learning to hit a nail with a hammer), to being highly social-emotional with fewer demands for coordination (e.g. smiling mutually). It seems likely that those tasks which are more socio-emotional require greater executive control (e.g. more inhibition and judgement in timing). It therefore follows that within a hierarchically ordered sensorimotor control network, action planning ranges from being highly subject to emotional modulation and executive control, to being more dependent on visuospatial control and sub-cortical motor planning. This range may reflect developmentally distinct mechanisms with visuospatial tasks requiring a lower level of hierarchical control than that serving more cognitive planning tasks (van Swieten et al. 2010). If socio-emotional learning goes hand-in-hand with development of executive control (Devine et al. 2016), individual differences in empathic function will be determined by the ability to learn hierarchical control in the expression of socially communicative actions. This may, as suggested by Gu et al. (2015), will reflect sensitivity of one level in the hierarchy to modulation by another level. Therefore, a cognitive/empathic divide may further correspond to differences at a higher or lower level of planning and control.

These points suggest that the marked effects of autism status that we found with respect to those items that ask about the perception of actions in relationship to emotional states, though not in relation to action-imagery, may well reflect a problem in autism, not in encoding actions in themselves, but in representing them at a ‘program’ level in relationship to socio-emotional states, where the highest levels of cognitive control are required. This is consistent with the diagnostic criteria for autism spectrum disorder, which heavily reflect relative impairment in the awareness and intentional control of actions, especially those embodying socio-emotional states.

A potential objection to our proposal is that our questionnaire simply asks people about their ability to understand feelings. It may be suggested that an item such as “I tend to pick up on people’s body language”, which is the most discriminative item in the questionnaire, simply taps into interpersonal sensitivity. We offer two observations in response to this point. Firstly, we note the high internal consistency of the questionnaire and that many of the items are unlikely to tap into interpersonal sensitivity (e.g. “music that I like makes me want to dance”). Secondly, the feelings subscale of the AFQ on its own confines itself to questions about emotionally-laden actions without asking about social behaviour in general, and yet this scale retains the same capacity as the EQ questionnaire (and arguably improves upon it) of being highly discriminative between groups, whether divided by sex or autism status. This provides further evidence that empathy is closely associated with motor cognition. A third point is that our preliminary study using this questionnaire suggests that it taps into emotional awareness, especially given correlations with activity in somatosensory cortex (Williams et al. 2015).

In conclusion, the actions and feelings questionnaire is a brief, self-report questionnaire that was designed to assess motor cognition and which demonstrates marked effects of sex and autism status. This provides further evidence that the empathic problems that occur in autism are closely related to variation in motor cognition, particularly through the awareness and control of higher level actions embodying socio-emotional states, which could underpin a wide range of the symptoms associated with autism spectrum conditions.

## Electronic Supplementary Material

Below is the link to the electronic supplementary material.

Supplementary material 1 (XLS 717 KB)
